# Neurosurgical Biopsy and Resection for Diagnosis and Treatment of *Balamuthia mandrillaris* Amebic Encephalitis, United States

**DOI:** 10.3201/eid3207.260725

**Published:** 2026-07

**Authors:** Beenish Rubbab, Ammar Adenwalla, Natasha Spottiswoode, Julia C. Haston, Sarah Firmani, Sumit Singh, Veena Rajaram, Jay Ramos, Ibne Karim M. Ali, Brett Whittemore, Natasha W. Hanners

**Affiliations:** University of Texas Southwestern Medical Center, Dallas, Texas, USA (B. Rubbab, A. Adenwalla, S. Singh, V. Rajaram, B. Whittemore, N.W. Hanners); University of California San Francisco, San Francisco, California, USA (N. Spottiswoode); Centers for Disease Control and Prevention, Atlanta, Georgia, USA (J.C. Haston, I.K.M. Ali); Children’s Health, Dallas (S. Firmani, J. Ramos)

**Keywords:** Balamuthia mandrillaris, parasites, ameba, meningitis/encephalitis, nitroxoline, infectious encephalitis, United States

## Abstract

We report a systematic case review of antemortem neurosurgical resections and biopsies and outcomes including new lesions after procedure and survival in *Balamuthia mandrillaris* granulomatous amebic encephalitis. The investigation was prompted by a 5-year-old patient in the southwestern United States who was treated with nitroxoline, the 2021 Centers for Disease Control and Prevention regimen, and underwent 2 resections; initial resection site recurrence and a new lesion after resection prompted the question whether complete resection versus biopsy is associated with better outcomes. We conducted a literature review and found no substantial difference between neurosurgical resection versus biopsy-only groups. Limitations include case review, number of cases, and incomplete data available. Additional analyses comparing neurosurgical outcomes with outcomes of those diagnosed via blood or cerebrospinal fluid and metagenomic next-generation sequencing might provide more definitive answers. This case and systematic review provide evidence that treatment with nitroxoline and neurosurgical resection could contribute to survival in *Balamuthia* encephalitis case-patients.

Free-living amebae (FLA) are soil- and water-dwelling unicellular organisms found throughout the world that cause rare but often fatal infections (*1*,*2*). *Acanthamoeba* and *Balamuthia* are 2 genera of FLA that cause granulomatous amebic encephalitis (GAE), a subacute disease characterized by focal neurologic deficits, altered mental status, and >1 parenchymal brain lesions on imaging. The true incidence is unclear because of diagnostic and reporting limitations, but in the United States, <20 cases are reported annually; however, >90% of infected patients who have central nervous system (CNS) involvement die (*1*,*2*).

*Acanthamoeba* spp. FLA primarily affect immunocompromised hosts, whereas *Balamuthia mandrillaris* amebae also infect immunocompetent hosts. *Balamuthia* spp. amebae exist as environmentally stable cysts and infectious trophozoites, entering the body through the respiratory tract or open skin wounds and spreading hematogenously to the organs, most notably the brain (*2*–*9*). *Balamuthia* was first identified in a pregnant mandrill (*Papio sphinx*) in 1990, but as a result, posthumous human diagnoses were made dating back to 1974 (*10*). Since then, *B. mandrillaris* GAE cases have been diagnosed worldwide, many among children, and often with fatal outcomes. A review of 109 US cases during 1974–2016 revealed a 90% mortality rate (*2*). Even when infections are diagnosed antemortem and patients receive antiamebic medications, the fatality rate exceeds 75% (*11*).

The Centers for Disease Control and Prevention (CDC) recommends a regimen for *B. mandrillaris* GAE including pentamidine, sulfadiazine, azithromycin or clarithromycin, a triazole, flucytosine, and miltefosine (*12*). In 2025, CDC added nitroxoline to the recommended regimen (*12*). A study published in 2018 screened 2,177 clinically approved compounds (including the CDC-recommended regimen) for in vitro activity against *B. mandrillaris* amebae (*13*)*.* A quinoline antibiotic, nitroxoline, was found to be the most potent and selective of all agents tested (including the drugs in the recommended regimen) against both cystic and trophozoite forms and at pharmacologically relevant concentrations (*13*). Nitroxoline has been used with a favorable side effect profile for human urinary tract infection treatment since the 1970s, including in pediatric patients (*14*). In 2021, an adult patient treated with nitroxoline for *Balamuthia* GAE survived (*15*). Herein, we describe a pediatric patient successfully treated with a combination of nitroxoline, the 2021 CDC recommended regimen, and 2 neurosurgical resections. We also conducted a literature review of previously published cases to determine any difference between neurosurgical resection versus biopsy-only groups.

## Case Report

**Figure 1:**
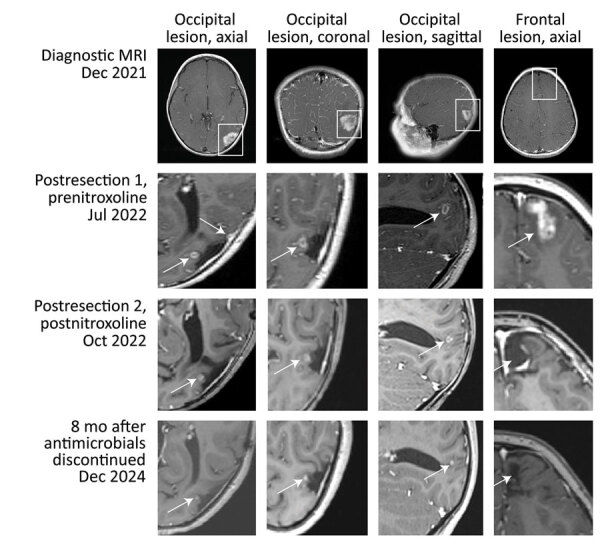
Axial, coronal, and sagittal postcontrast T1-weighted MRI images from study of neurosurgical biopsy and resection for diagnosis and treatment of *Balamuthia mandrillaris* amebic encephalitis, United States. Top row images show patient’s primary occipital and frontal lesions (boxes). Three weeks after resection of the first occipital lesion (second row), recurrent nodules (arrows) developed around the resection cavity. Before nitroxoline initiation, those nodules were enlarging. MRI in July 2022 (second row) showed a new, 7-mm diameter, indistinct, fluid attenuated inversion recovery–hyperintense, enhancing focus in the anterior aspect of the left superior frontal gyrus. After nitroxoline (third row), the occipital lesions retracted (arrows) and were no longer ring-enhancing. No recurrent lesions were noted at the resection site of the frontal lesion, which was resected while the patient was on multidrug therapy and 4 weeks before starting nitroxoline. Follow-up imaging 8 months after antimicrobial treatment was discontinued (fourth row), a stable, residual nodule (arrows) in the medial resection cavity remained, thought to represent gliosis. MRI, magnetic resonance imaging.

In autumn 2021, a previously healthy 5-year-old was brought to care with new-onset seizures and headaches. Persisting symptoms prompted brain magnetic resonance imaging (MRI), revealing a heterogeneously enhancing cortical mass at the junction between the left temporal and occipital lobes with a large region of surrounding T2 fluid attenuated inversion recovery (FLAIR) hyperintensity (Figure 1).

**Figure 2:**
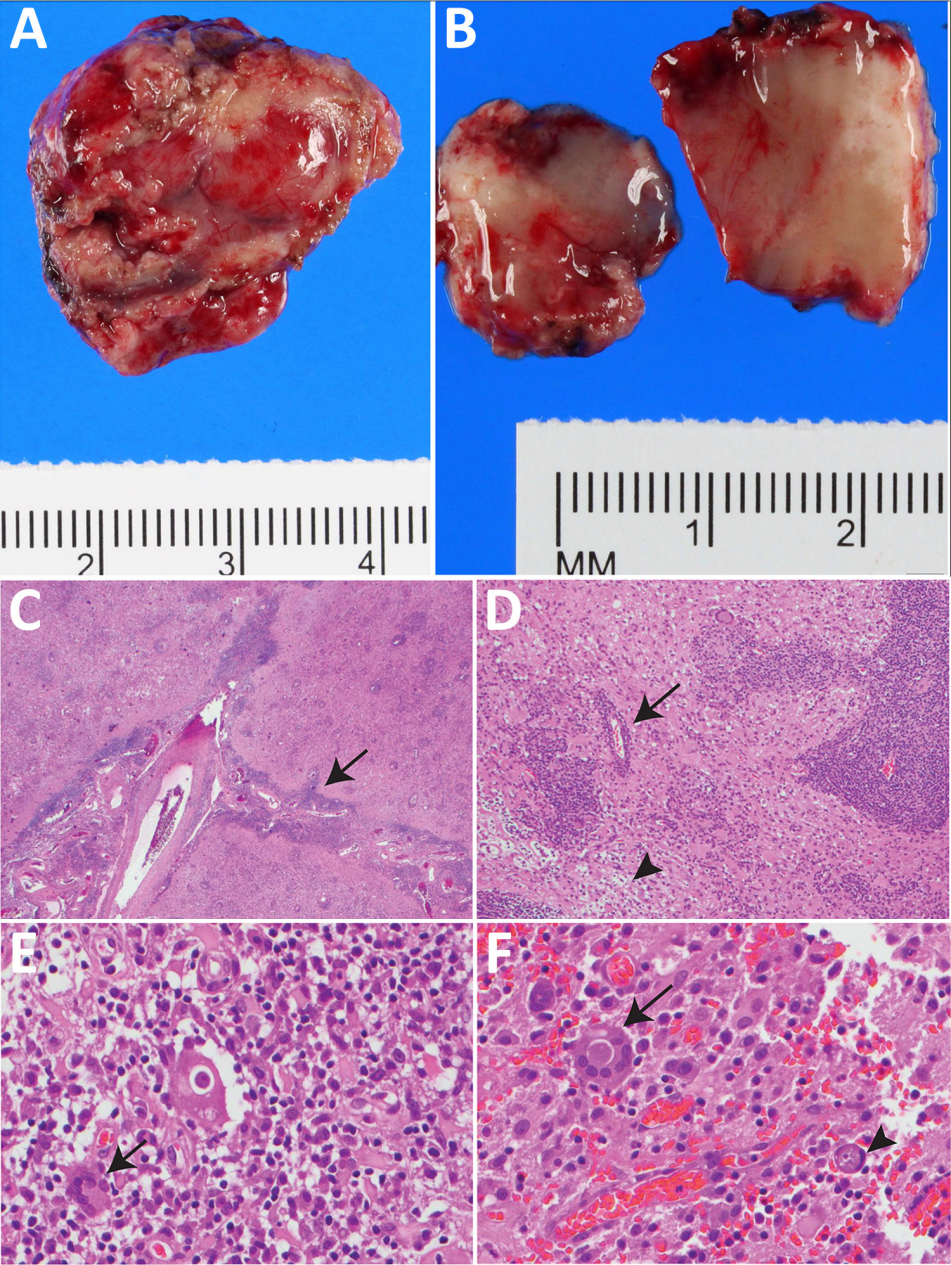
Resected occipital lesion gross and histopathology from patient in study of neurosurgical biopsy and resection for diagnosis and treatment of *Balamuthia mandrillaris* amebic encephalitis, United States. A) Gross pathology revealed pink-tan, firm tissue fragments. B) Cut sections reveal a heterogenous, tan-pink, and soft surface with effacement of the white and gray matter demarcation. C, D) Hematoxylin and eosin stained sections of the brain show leptomeningeal thickening with striking perivascular lymphocytic infiltrate (C, arrow) extending into the cortical parenchyma with foci of lymphocytic infiltration into the vessel wall, consistent with vasculitis (D, arrow). The architecture of the cortical gray matter is obliterated by neuronal loss, reactive gemistocytic astrocytes and infiltration by macrophages, multinucleated giant cells, and lymphocytes. This involves the adjacent white matter with areas of parenchymal loss/liquefactive necrosis (D, arrowhead). Original magnification ×2 for panel C, ×10 for panel D. E, F) Many of the multinucleated giant cells (E, arrow) contain large round thick-walled parasites (F, arrow) containing granular material and an occasional inconspicuous, dot like nucleus (F, arrowhead). Original magnification ×40 for panels E and F. Occasional structures resembling engulfed organisms are seen in the parenchyma (D–F).

With malignant tumor on the differential diagnosis, the patient underwent left occipital craniotomy and the specimen was removed en bloc and sent to pathology. The neurosurgeon chose resection margins on the basis of intraoperative frameless stereotactic navigation and the appearance and feel of normal-appearing, soft, edematous brain. Postoperative MRI confirmed an enhancing mass with no change in the surrounding edema. The neurosurgery team consulted the pediatric infectious diseases service because the preliminary pathology report was concerning for infection rather than tumor. We suspected *Balamuthia* GAE because of course chronicity, immunocompetent host, and extensive soil exposures, including making mudpies. However, we also considered in the initial consultation *Acanthamoeba* (considered less likely because the patient was immunocompetent), *Naegleria* (considered less likely because the patient had no typical acute manifestations), mycobacterial diseases including nontuberculous (NTM) and tuberculosis (TB) (considered less likely because the patient was immunocompetent [NTM] and had no specific exposure [TB]), and endemic fungal infections with spread to the CNS (again considered less likely because the patient was immunocompetent). Histopathology revealed leptomeningeal lymphocytic infiltrate extending into the cortical parenchyma with focal vasculitis, along with lymphocytes and multinucleated giant cells containing large, round, thick-walled parasites, consistent with GAE (Figure 2). Special tissue stains were negative for acid-fast bacillus and fungal elements, but a sample of brain tissue sent to CDC’s Free-living and Intestinal Amebas laboratory (National Center for Emerging and Zoonotic Infectious Diseases, Division of Foodborne, Waterborne, and Environmental Diseases) was positive for *B. mandrillaris* amebae by PCR (*24*).

**Figure 3:**
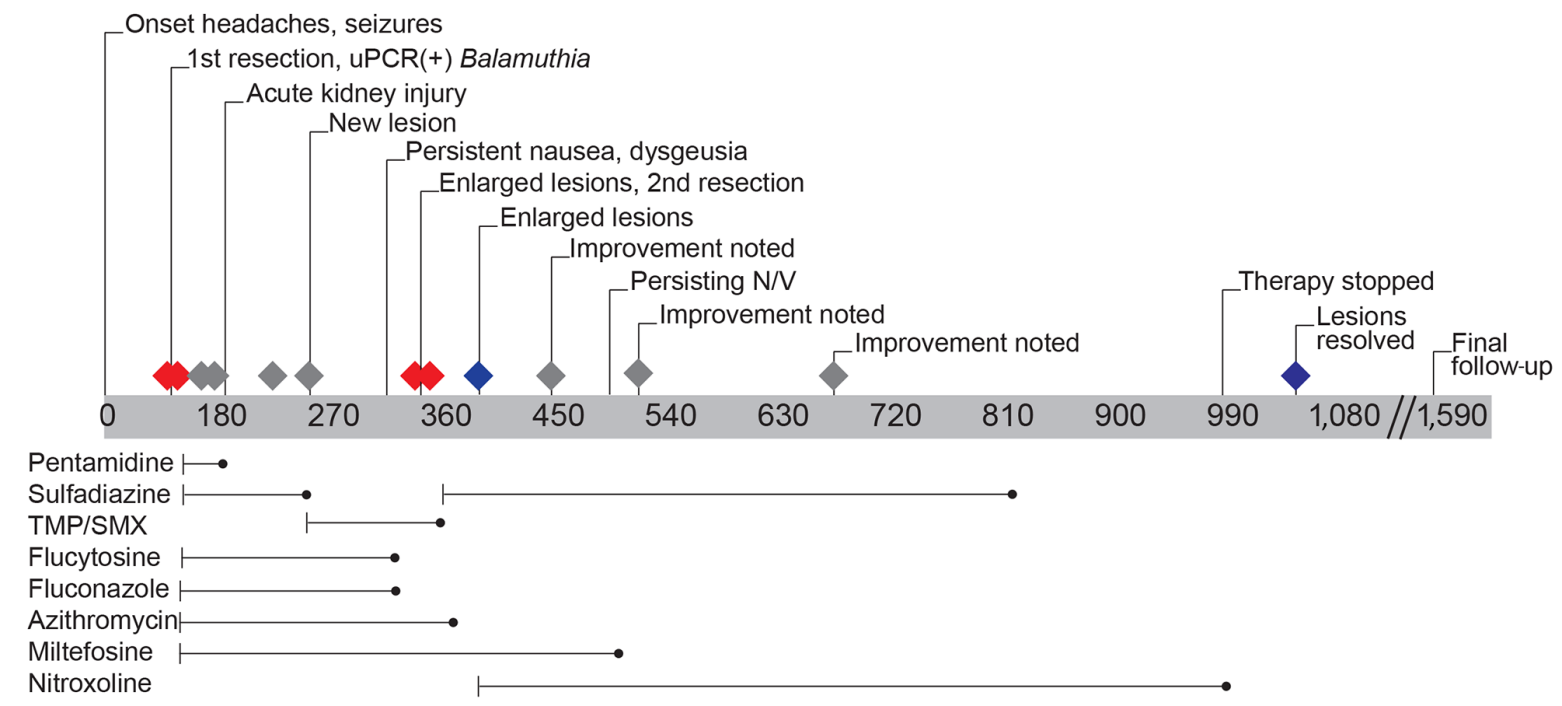
Timeline of events and medications for patient in study of neurosurgical biopsy and resection for diagnosis and treatment of *Balamuthia mandrillaris* amebic encephalitis, United States. Gray bar shows days since initial evaluation; red diamonds indicate magnetic resonance imaging (MRI) obtained before and after surgical dates; gray diamonds indicate interval MRI; blue diamonds indicate MRI before and after end date of nitroxoline. Nitroxoline dosing was titrated over 1 week to a total of 30 mg/kg/day 3 times/day; other medication dosing was pentamidine, 4 mg/kg (80 mg) every 24 hours; sulfadiazine, 50 mg/kg (1,000 mg) every 6 hours; azithromycin, 20 mg/kg (400 mg) every 24 hours; fluconazole, 12 mg/kg (240 mg) every 24 hours; flucytosine, 37.5 mg/kg (750 mg) every 6 hours; and miltefosine, 2.5 mg/kg (50 mg) every 24 hours. Pentamidine was discontinued after 8 weeks because of concern for renal toxicity. Sulfadiazine was briefly switched to trimethoprim/sulfamethoxazole because of manufacturer supply shortage and ultimately discontinued because of cost and administration concerns. Because of persistent nausea, vomiting, dysgeusia, and anorexia, flucytosine, fluconazole, azithromycin, and miltefosine were each discontinued, and the patient remained on nitroxoline for a total of 19 months. N/V, nausea and vomiting; TMP/SMX, trimethoprim/sulfamethoxazole; uPCR, universal broad-range PCR amplicon sequencing.

On the basis of CDC recommendations at diagnosis, the patient was started on pentamidine (4 mg/kg [80 mg] every 24 h), sulfadiazine (50 mg/kg [1,000 mg] every 6 h), azithromycin (20 mg/kg [400 mg] every 24 h), fluconazole (12 mg/kg [240 mg] every 24 h), flucytosine (37.5 mg/kg [750 mg] every 6 h), and miltefosine (2.5 mg/kg [50 mg] every 24 h) (Figure 3). On this regimen, 5 months later, new frontal headaches developed. Repeat MRI at that time showed a new, 7-mm diameter, enhancing focus in left superior frontal gyrus and 3 enhancing nodules at the peripheral resection margins of the primary left occipital site. The multidrug regimen was continued, and repeat MRI 2 months later showed interval increase in the size of the left frontal lesion and surrounding T2/FLAIR hyperintensity (Figure 1). The patient subsequently underwent a left frontal craniotomy for en bloc resection using intraoperative frameless stereotactic navigation. Intraoperative ultrasound revealed no residual hyperechoic tissue. Pathology resembled first resection, and the tissue was again positive for *B. mandrillaris* amebae by PCR testing at CDC.

Given concern for progressive disease on existing therapy, along with promising in vitro data and a published successful case report (*13*,*15*), we decided to add nitroxoline to the regimen. We obtained Institutional review board approval, Food and Drug Administration emergency investigational drug designation, and parental consent. After emergency designation, we identified a foreign nitroxoline manufacturer. However, because the patient’s imaging and clinical status were worsening and obtaining the drug directly from the manufacturer would have meant a delay of several weeks, to ensure timely administration, we procured a small supply from the same manufacturer via University of California San Francisco Investigational Drug Services. The patient received the first dose of nitroxoline 1 month after second resection. We sourced subsequent treatment directlyfrom the manufacturer and established a formal contractual agreement to secure ongoing access to the investigational agent.

While monitoring the patient in the hospital for both medication side effects and worsening symptoms, we titrated the nitroxoline dose over 1 week to a total of 30 mg/kg/day (3×/d). We chose that dose because it is the maximum pediatric dose described in published nitroxoline reviews and was comparable to dosing used for an adult *B. mandrillaris* patient treated with nitroxoline (*14*,*15*). Clinical, laboratory, electrocardiogram, and dental monitoring showed that the patient tolerated nitroxoline without substantial side effects.

MRI at 6 weeks after nitroxoline initiation showed considerable improvement in the ring-enhancing lesions at the margin of the left occipital surgical cavity, no recurrence of left frontal lesion, and no new lesions. Her next MRI scans, at 3 and 9 months after nitroxoline initiation, showed continued improvement in the ring-enhancing lesions of the occipital region and no new lesions. MRI 1 year after nitroxoline initiation showed expected encephalomalacia and gliosis at previous resection cavities, resolution of ring-enhancing occipital lesions, and no new lesions. In total, the patient was treated for 2 years and 4 months with antiamebic therapy, including nitroxoline for the final 19 months of therapy. At 8 months after therapy ended, a tiny residual nodule in the medial resection cavity was stable and thought to represent gliosis (Figure 1). Clinical follow-up 4 years after diagnosis revealed a healthy, active child without focal neurologic findings but with mild neurocognitive and learning delays on neuropsychiatric testing.

## Systematic Literature Review

The progression of disease and need for a second resection in this case raised the question whether complete resection, versus biopsy only, might be beneficial to outcomes of *B. mandrillaris* GAE. We hypothesized that because of the prevalence of vasculitis in and proposed hematogenous spread of *Balamuthia* disease, biopsy might promote spread by disruption of the blood–brain barrier and transection of affected vessels, whereas complete resection, aided by reducing the burden of organisms, could be associated with better outcomes. To evaluate that possibility, we performed a systematic review of the literature according to the guidelines for Preferred Reporting Items for Systematic Reviews and Meta-Analyses (*16*,*17*). We searched for published articles via PubMed (last reverified 2026 Jun 4) to identify cases in which *B. mandrillaris* GAE was diagnosed using specimens obtained antemortem via neurosurgical procedures, including complete resection (group 1) or subtotal resection or biopsy (group 2). We used the following search terms: Balamuthia AND (biopsy OR neurosurgery OR neurosurgical OR excision OR resection OR brain mass OR tumor). That initial search returned 231 articles (Appendix 1 Table 1). We then filtered the list by English language to 223 articles and then further filtered for human cases, bringing the number of articles to 161. We also found 6 published articles not captured in the PubMed search (*15*,*18*–*23*); all were noted in the course of reviewing other articles and considered pertinent to this topic. However, noting the absence of those articles in the PubMed search, to avoid omission of articles or bias, we reviewed the nonhuman filtered cases to identify erroneously omitted cases and conducted another search using terms “Balamuthia AND case report.” Those searches identified no additional articles that did not meet exclusion criteria. In total, we reviewed 168 articles (Appendix 1 Table 2).

This study was confined to antemortem neurosurgical procedures only because of the hypothesis that cutting into diseased tissue without (presumptively) substantially diminishing the burden of ameba might worsen disease outcomes in *Balamuthia* GAE. Therefore, we excluded the following types of cases: no neurosurgical specimen obtained antemortem (n = 36), only cutaneous disease (n = 3), infection not verified by the definitions we devised or cases of other FLA (n = 3), and co-infections (which might alter the pathogenesis) (n = 2). We also excluded the following types of studies: primary purpose of subject review (n = 30), in vitro studies (n = 22), primary purpose to report specimen data or imaging findings without substantial clinical descriptions (n = 7), environmental specimens (n = 5), duplicated cases (n = 4), editorials (n = 2), mouse studies (n = 1), nonhuman cases (n = 1), retracted articles (n = 1), articles for which we could not obtain the full text (n = 1), or case reports that did not include the targeted data (n = 1) (Appendix 1 Table 3). 

We defined a confirmed case of *B. mandrillaris* GAE as a patient with clinical encephalitis with imaging findings supportive of diagnosis and either molecular diagnostics (PCR or metagenomic next-generation sequencing; Karius, https://kariusdx.com) or immunofluorescence or immunohistochemistry staining positive on pathologic specimens for *B. mandrillaris.* Cases that had only positive serologic results or morphologic evidence of ameba were not considered diagnostic for *B. mandrillaris* GAE.

We defined resection cases as cases in which the article defined the procedure as resection or complete excision or en bloc in an antemortem neurosurgical procedure. We defined biopsy cases as cases in which the article reported biopsy, excisional biopsy, or subtotal resection of the lesion in an antemortem neurosurgical procedure. Cases involving only blood, cerebrospinal fluid (CSF) specimens, or skin or other organ biopsies were outside the scope of this study.

We reviewed and extracted data from the 68 articles meeting inclusion and exclusion criteria. Of those, 20 were resection cases; data collected included clinical manifestation description (country of residence, age, sex, initial symptoms, notable medical history), number and location of lesions at evaluation, site of lesion resected, antiamebic medications (if used and with details to the extent published), presence of vasculitis on biopsy, additional lesions identified after procedure, and use of steroids before *Balamuthia* diagnosis. The remaining 48 articles were on biopsy cases; data collected included clinical manifestation description, whether >1 lesion was present at diagnosis, whether additional lesions were identified after procedure, and whether steroids were used before *Balamuthia* diagnosis.

For resection and biopsy studies including resection cases, we recorded the number that had additional lesions identified on brain imaging (most advanced obtained, either computed tomography or MRI) after the antemortem neurosurgical specimen collection. We also recorded the number for whom steroids were reported to be given and the number who survived the infection. For the steroid use and survival analyses, we calculated percentages on the basis of the total number of resection cases or biopsy cases. For the lesion number analysis, the denominator was the number of cases for which information was available by imaging reports, because some patients died before follow-up imaging after the procedure could be obtained.

We used GraphPad Prism (https://www.graphpad.com) for statistical analysis. We examined for statistical differences between resection cases and biopsy cases using Fisher exact tests.

## Results

From our systematic review of the literature, plus the case we report here, we found 7 published articles describing 1 or 2 cases of *B. mandrillaris* intracranial lesions that were surgically resected and the patient survived (*18*,*19*,*25*–*29*). Of 13 cases in which the patient subsequently died, each was associated with >1 of the following: multiple CNS lesions at initial evaluation (n = 5) (Appendix 2 Table 1 references 5,17–19), advanced neurologic symptoms (i.e., hemiparesis, seizures, signs of increased intracranial pressure or herniation, loss of consciousness) (n = 9) (Appendix 2 Table 1 references *5,6,10,13,17–19*), vasculitis on imaging or pathology (n = 5) (Appendix 2 Table 1 references *5–7,10,17)*, a regimen distinct from the 2021 CDC medication regimen (<4 agents used) (n = 11) (Appendix 2 Table 1, references *5–7,12,13,16–19*), or steroid use preceding diagnosis and antiamebic treatment (n = 6) (Appendix 2 Table 1 references *5,10,12,17-19*).

We questioned whether resection, either subtotal or biopsy or complete resection, increased likelihood of patient death or additional lesions arising after the neurosurgical procedure. Vasculitis is a prominent feature of *B. mandrillaris* GAE (*30*). We hypothesized that incision into infected tissue might promote spread of ameba if total resection is not achieved. Therefore, we conducted an additional review of the cases that had biopsy or subtotal resections for outcomes of survival or death and whether additional lesions were discovered on follow-up imaging after the procedure. We found no statistical difference between the groups, however (Table; Appendix 2 Table 2), and similar rates of steroid use before *B. mandrillaris* GAE diagnosis.

**Table:** Analyses of outcomes between resection and biopsy groups in study of neurosurgical biopsy and resection for diagnosis and treatment of *Balamuthia mandrillaris* amebic encephalitis, United States

Category	No. (%) cases*		Statistically significant difference between groups†
	Resection, n = 20	Biopsy, n = 48	
New lesions after procedure			
Y	9 (45)	17 (35)	N (p = 0.75)‡
N	6 (20)	9 (19)	
Not clear	5 (25)	22 (46)	
Survival			
Y	7 (35)	8 (17)	N (p = 0.12)
N	13 (65)	40 (83)	
Steroids			
Y	9 (45)	27 (56)	N (p = 0.43)
Not reported	11 (55)	21 (44)	

*Includes case from this study. †For yes/no responses only. Method used Fisher exact test. ‡For new lesions, the “not clear” cases were omitted from statistical analysis.

## Discussion

We report the excellent outcome for a pediatric patient with *B. mandrillaris* GAE treated with nitroxoline. Factors contributing to GAE survival are not clear, but we believe that nitroxoline and neurosurgical interventions were important to this patient’s survival. A multidisciplinary team, including pharmacy, psychology, and child life services, individualized the patient’s complex medication schedule, which, along with family vigilance, undoubtedly contributed to the success of this regimen. Our findings of clinical and radiographic improvement add to a growing body of literature suggesting that nitroxoline, in combination with other antiamebic drugs and surgical resection, could improve outcomes from this rare, highly fatal disease. Nitroxoline has better tolerability and in vitro efficacy against *B. mandrillaris* infection than other antiamebic agents (*13*,*31*). In a review of safety and efficacy of nitroxoline in UTI treatment, only 9.8% of patients reported adverse effects (primarily nausea) (*13*,*14*,*31*). In contrast, the standard regimen, which includes pentamidine, miltefosine, a triazole, flucytosine, azithromycin, and sulfadiazine, has substantial toxicities, often limiting use and leading to other adverse conditions. Indeed, acute kidney injury and hypertension occurred in this patient, but resolved after discontinuation of pentamidine. In addition, gastrointestinal side effects of dysgeusia, anorexia, nausea, vomiting, and diarrhea necessitated placing a gastrostomy tube for nutrition; those symptoms resolved after sequential discontinuation of miltefosine, fluconazole, flucytosine, and azithromycin and did not recur during nitroxoline monotherapy.

Nitroxoline was continued throughout the final 19 months’ duration of this patient’s antiamebic therapy, and clinical and radiographic improvement continued even during the final 6 months on nitroxoline monotherapy, suggesting that nitroxoline could be a contributor to her survival. Our review of the literature subsequent to this case identified 2 other reported uses of nitroxoline, in addition to the adult case reported in the introduction (*15*). Those 2 cases were pediatric as well, but neither included neurosurgical resection. In the first case, a 4-year-old had *B. mandrillaris* infection diagnosed by Karius testing of blood and confirmed by CSF PCR. Nitroxoline was started 1 month into diagnosis, but the patient died 1 month later (*32*). The other pediatric case, in a 2-year-old, was diagnosed by biopsy and was included in this systematic review; the patient survived, as reported at 1 year after manifestation (*33*).

We cannot definitively determine the independent contribution of nitroxoline to treatment success on the basis of the case we report or the few others that have been published. However, a lack of recurrence or progression over a period of 19 months on nitroxoline, including 6 months of nitroxoline alone, supports the possibility that nitroxoline contributed to recovery. Before nitroxoline was initiated, testing showed a new frontal lesion and progression of that lesion (Figures 1, 3), but after nitroxoline treatment began, no new progression or lesions developed, and occipital residual lesions shrank. In addition, at the resection margins of the frontal lesion addressed in the second resection, surrounding residual nodules did not develop during nitroxoline treatment, unlike after the first resection before nitroxoline treatment. Variations in the success of complete resection microscopically, brief steroid use postoperatively after first resection, and being on any antiamebic therapy at the second resection all likely contributed as well.

The role of neurosurgical intervention for *Balamuthia* infections remains unclear. Whereas Karius testing and metagenomic next-generation sequencing can provide new opportunities for diagnosis without neurosurgical intervention (*20*,*32*,*34*–*42*), brain biopsy can lead to definitive diagnosis and early therapy. However, few cases manifest with lesions amenable to complete resection (*18*,*19*,*25*–*29*). Lesion recurrence after this patient’s first surgery, despite using the CDC-recommended regimen, suggests that infection might have extended into normal-appearing tissue, beyond resection margins (i.e. incomplete resection); the previous CDC-recommended regimen could be insufficient to treat *Balamuthia* GAE; or vasculitis or resection in the setting of steroids or vasculitis without concurrent medication against *Balamuthia* amebae might contribute to dissemination (*30*). Of interest, we saw no recurrence from the patient’s second resection, but at that time, the patient had concurrent treatment with antiamebic medications, including nitroxoline shortly thereafter. Our review and analyses suggest that variations in completeness of excision might not determine the recurrence or development of new lesions because no substantial difference in new lesions after biopsy or resection were noted between the 2 groups. We did find a trend toward association of excision with survival that warrants further investigation; however, the data did not show a statistically significant survival benefit to excision.

Our study is limited by the nature of literature reviews, including incomplete information and relatively small numbers, given the rarity of the disease. For example, with respect to steroid use, some articles (3 cases of the biopsy-only group) reported that the patients were initially treated for CNS TB. Providing steroids is the standard of care for such cases, so the lack of steroids reported in many of those cases exemplifies possible missing data in the literature (*43*). Future studies comparing the outcomes of patients who have antemortem neurosurgical resections or biopsies with those who only have non-CNS procedures for diagnosis or therapy (i.e., skin biopsies, blood or CSF specimens only) will be further informative on the risks of neurosurgical intervention in *Balamuthia* GAE. In addition to that theorized risk, risks of neurosurgery must always be considered before surgical intervention, but lack of recurrent or new lesions in this patient case report after the second resection might have been the result of the multidrug regimen used before resection, and the addition of nitroxoline shortly after resection.

In this systematic review and analysis, we found no statistically significant outcomes between the group for whom biopsy was conducted for diagnosis and the group for whom complete resection for diagnosis and therapy was used. However, we did find a trend toward benefit in survival in complete resection group that warrants further study. 

In conclusion, we report a systematic review of survival in *B. mandrillaris* GAE after antemortem neurosurgical resections and biopsies, including successful treatment of a pediatric patient in the southwestern United States. For patients with *B. mandrillaris* GAE, neurosurgical management, multidrug therapy, and nitroxoline treatment could improve outcomes associated with this deadly infection.

Appendix 1 Literature review details for neurosurgical biopsy and resection for diagnosis and treatment of *Balamuthia mandrillaris* amebic encephalitis, United States.

Appendix 2 Additional information for neurosurgical biopsy and resection for diagnosis and treatment of *Balamuthia mandrillaris* amebic encephalitis, United States.
