# Supplementary material for: Neurosurgical Biopsy and Resection for Diagnosis and Treatment of Balamuthia mandrillaris Amebic Encephalitis, United States
Source: Emerg Infect Dis. 2026 Jul;32(7):1065–73. doi: 10.3201/eid3207.260725 (PMC13322428; doi:10.3201/eid3207.260725)
Supplement: Appendix 2 — Additional information for neurosurgical biopsy and resection for diagnosis and treatment of Balamuthia mandrillaris amebic encephalitis, United States. [file 26-0725-Techapp-s2.pdf]

Article DOI: https://doi.org/10.3201/eid3207.260725

EID cannot ensure accessibility for supplementary materials supplied by authors. Readers who have difficulty accessing supplementary content should contact the authors for assistance.

Neurosurgical Biopsy and Resection for Diagnosis and Treatment of *Balamuthia mandrillaris* Amebic Encephalitis, United States

Appendix 2

Appendix 2 Table 1. A literature review of cases of *Balamuthia mandrillaris* granulomatous amebic encephalitis treated with surgical resection

Citation	Country	Age, Sex	Symptoms	Location of brain lesions (#)	Reported Vasculitis	Antiamebic medications (with dose and route if reported in publication)	Reported steroid use prior to Balamuthia diagnosis	Resected lesion	Additional lesions after procedure	Outcome (survival follow up months)
This case	USA	5 yo F	4 months of headache, seizures	Left occipital (1), left frontal (1)	Yes	See case report section	Yes	Left occipital (1), left frontal (1)	Yes, after first resection; no after second	Survived (4.5 years since diagnosis)
Mei et al. Clin Neurol Neurosurg. 2025 (*1*,*2*)	China	3 yo M	Slurred speech, seizures, fever	Left frontal	No	Miltefosine, fluconazole, rifampicin, albendazole, amphotericin B	Yes	Left frontal	No	Survived (1 mo)
Chen et al. BMC Neurol. 2025 (*3*)	China	9 yo M	Two days of dizziness treated initially as CNS tuberculosis	Right occipital lobe	No	Lincomycin, rifampicin, sulfamethoxazole, fluconazole, albendazole and gamma IFN	No	Right occipital lobe	Yes	Survived (24 months)
Zhang et al. Front Immunol. 2025 (*4*)	China	37 yo M	Right lower extremity lesion x6 mo, p/w dizziness, headache and AMS, treated initially as CNS tuberculosis x2 months	Left frontal lobe	No	Fluconazole 400 mg q day<br>Flucytosine 1.5 g QID<br>Clarithromycin 500 mg BID<br>Miltefosine 50 mg PO q8h<br>Albendazole (dose unclear) PO q12h<br>Pentamidine 300 mg IV q day	No	Left frontal	Yes	Death
Teng et al. Int J Infect Dis. 2025 (*5*)	China	10 mo old M	Fever, seizures, AMS	Frontal, temporal, parietal, occipital	Yes	Azithromycin, albendazole, TMP/SMX, fluconazole and flucytosine	Yes	Right parietal	Not clear	Death

Citation	Country	Age, Sex	Symptoms	Location of brain lesions (#)	Reported Vasculitis	Antiamebic medications (with dose and route if reported in publication)	Reported steroid use prior to Balamuthia diagnosis	Resected lesion	Additional lesions after procedure	Outcome (survival follow up months)
				lobes, basal ganglia, thalamus						
Javed et al. Surg Neurol Int. 2024 (*6*)	Pakistan	33 yo F	Back pain, headache, vomiting	Right frontotemporal	Yes	Azithromycin, meropenem, metronidazole, fluconazole, co-trimoxazole, rifampicin (all IV), miltefosine, amphotericin B (IT)	No	Right frontotemporal	Yes	Death
Liu et al. BMC Infect Dis. 2023 (*7*)	China	61 yo M	Headache	Right frontal	Yes	Azithromycin, fluconazole, flucytosine, TMP-SMX	No	Frontal	Not clear	Death
Peng et al. BMC Infect Dis. 2022 (*8*)	China	54 yo M	Numbness, weakness, headache, diplopia, ataxia	Right parietal (1), left occipitoparietal (1)	No	TMP-SMX 960 mg (160 mg TMP) PO BID<br>Azithromycin 500 mg PO daily<br>Flucytosine 2500 mg IV q12h<br>Amphotericin B IV titrated to 30 mg daily<br>Fluconazole 600 mg IV daily (replaced amphotericin B after 1mo)<br>Final regimen:<br>TMP-SMX PO, azithromycin PO	No	Right Parietal; Left occipitoparietal	Yes	Survived (7 mo)
Cuoco et al. Pediatr Infect Dis J. 2022 (9)	U.S.A.	4 yo M	Agitation, irritability, GTC seizure	Right posterolateral parietal	No	Flucytosine 750 mg PO QID*<br>Miltefosine 50 mg PO BID*<br>TMP-SMX 100 mg PO TID*<br>Azithromycin 200 mg PO daily*<br>Fluconazole 200 mg PO daily*	No	Parietal	No	Survived (12 mo)
Shehab et al. J Pediatric Infect Dis Soc. 2018 (*10*)	U.S.A.	13 yo F	Abdominal pain and headaches progressing to left-sided weakness, slurred speech and vomiting	Right parietal	Yes	Miltefosine, fluconazole, flucytosine, azithromycin, and sulfadiazine	Yes	Parietal	Yes	Death
Lehmer LM et al. Dermatol Online J. 2017 (*11*)	U.S.A.	84 yo M	Nodular ulcerative skin lesion; followed by headache, confusion, aphasia, apraxia	Bilateral occipital	Skin - yes, brain - no	Azithromycin, flucytosine, sulfadiazine	No	One of two occipital masses	No	Survived (12 mo)

Citation	Country	Age, Sex	Symptoms	Location of brain lesions (#)	Reported Vasculitis	Antiamebic medications (with dose and route if reported in publication)	Reported steroid use prior to Balamuthia diagnosis	Resected lesion	Additional lesions after procedure	Outcome (survival follow up months)
Kobayashi et al. Pathology. 2015 (*12*)	Japan	57 yo F	Headache, dysarthria	Right frontal lobe	No	None	Yes	Frontal lobe	Yes	Death
Krasaelap et al. Korean J Parasitol. 2013 (*13*)	Thailand	4 yo F	Headache, vomiting, ataxia; presented with obstructive hydrocephalus, tonsillar herniation	Right cerebellar hemisphere	No	Pentamidine 4 mg/kg/day<br>Sulfasalazine 200 mg/kg/day<br>Fluconazole 12 mg/kg/day<br>Clarithromycin 14 mg/kg/day<br>Amphotericin B 1 mg/kg/day	No	Cerebellar	No	Death
Doyle et al. J Neurosurg. 2011 (*14*)<br><br>Botterill et al. Clin Neurol and Neurosurg. 2011 (*15*)	Australia	80 yo F	Skin plaques and nodules on the hand, followed by GTC seizures, disorientation, and seizure 1 mo later	Right medial frontal	No	Pentamidine 300 mg IV daily<br>Azithromycin 600 mg PO daily<br>Itraconazole 200 mg PO BID<br>Sulfadiazine 1500 mg PO QID<br>Flucytosine 1000 mg PO TID<br>Amphotericin B liposomal 3 mg/kg IV daily replaced pentamidine for 4 weeks<br>Final regimen: Azithromycin, itraconazole, sulfadiazine, flucytosine (7 mo total)	Yes	Right medial frontal	No	Survived (18 mo)
Sarica et al. Turkish Neurosurg. 2009 (*16*)	Turkey	75 yo M	Headaches, speech problems and nervousness	Left temporal	No	Amphotericin B IV 1.5 mg/kg/day divided BID, followed by Amphotericin B liposomal 200 mg IV daily<br>Miconazole 350 mg/m2/day IV divided TID	No	Left temporal abscess drainage with capsule resection	No	Death
Tavares et al. J Clin Microbiol. 2006 (*17*)	Portugal	8 yo M	Progressive headaches, diplopia, vomiting, lethargy, papilledema	Right frontal and right temporal	Yes	Fluconazole (10 mg/kg)<br>Trimethoprim-sulfamethoxazole (15 mg/kg)<br>Rifampin (10 mg/kg)	Yes	Right frontal	Yes	Death
Galarza et al. Pediatr Neurol. 2002 (*18*)	Argentina	12 yo M	Non-caseating skin lesions followed by fever, lethargy, hemiparesis	Cerebral, brainstem and cerebellum (many)	No	Pentamidine, flucytosine, and fluconazole ("usual doses")	Yes	Cortical mass	Not clear	Death
	Argentina	5 yo M	Chronic osteomyelitis and granulomatous skin lesion x1y, seizure, LOC	Cerebral, mostly frontal (many)	No	Pentamidine, flucytosine, fluconazole, and clarithromycin ("usual doses")	No	One frontal lesion	Not clear	Death

Citation	Country	Age, Sex	Symptoms	Location of brain lesions (#)	Reported Vasculitis	Antiamebic medications (with dose and route if reported in publication)	Reported steroid use prior to Balamuthia diagnosis	Resected lesion	Additional lesions after procedure	Outcome (survival follow up months)
	Argentina	3 yo F	Partial seizures, decreased LOC, left hemiparesis	Right frontal	No	Pentamidine, flucytosine, fluconazole, and clarithromycin (“high doses”)	No	Frontal lesion	Not clear	Death
Deol et al. Surg Neurol. 2000 (*19*)	U.S.A.	38 yo M	Skin nodules, ulcers for 6 mo, then seizures	Temporal (1), occipital (1)	No	No	Yes	Occipital lesion	Yes	Death

Abbreviations used: yo – year(s) old; mo – month(s); M – male; F - female; GTC – generalized tonic-clonic; CNS – central nervous system; PO - per oral; IV – intravenous; IT - intrathecal; q – every, d - day, BID – twice a day; TID – three times a day; QID – four times a day; TMP-SMX – trimethoprim-sulfamethoxazole; g – grams; mg – milligrams; kg – kilograms; mg/m2 – milligrams per square meter.
*Route of administration confirmed with corresponding author (J. Cuoco, pers. comm., email, 2026 Jan 8).

Appendix 2 Table 2. Brief summary of outcomes of *Balamuthia mandrillaris* granulomatous amebic encephalitis cases with subtotal resection or biopsy

Case No.	Citation	Clinical presentation	More than one lesion prior to biopsy	Steroids prior to antiamebic treatment	Antiamebic Treatment (with doses if described)	Increased # lesions after biopsy	Outcome (duration at follow up if given)
1	Degese et al. Acta Parasitol. 2025 (*20*).	36 yo M with behavior changes, disorientation, seizures, headache	Not clear	Not described	Not described	Not clear	Death
2		7 yo F headache and increased intracranial pressure and 3 years ago plaques on right upper extremity	Not clear	Not described	Not described	Not clear	Death
3		2 yo M with generalized tonic-clonic seizures	Not clear	Not described	Flucytosine, fluconazole, albendazole, miltefosine and nitroxoline (sulfadiazine discontinued due to neutropenia)	Not clear	Survived
4	Khurana et al. Indian J Med Microbiol. 2015 (*21*).	18 yo M with 2 weeks of headache diplopia, fever and vomiting x1 week	Yes	Not described (but treated for CNS TB prior to biopsy)	Albendazole, amphotericin B, clarithromycin	Not clear	Death
5	Griesemer et al. Pediatr Neurol. 1994 (*22*).	13 yo F with headache and diplopia and vomiting	Yes	Yes	None	Not clear	Death
6	Edminster et al. Acta Neuropathol Commun. 2025 (*23*).	58 yo F with 10 days of progressive right facial numbness, left-sided hemiparesis, imbalance, and tinnitus	No	Yes and cyclophosphamid e	None (broad-spectrum antimicrobials)	Yes	Death
7	Liang et al. Diagn Microbiol Infect Dis. 2025 (*24*).	49 yo F in China with right lower extremity cutaneous lesion 6 mo prior, followed by	Yes	Not described	Azithromycin (500mg, QD) Metronidazole (0.5g, Q8H) Flucytosine (1.5g, Q6H) Fluconazole (0.4g, QD)	Not clear	Survived (2 months)

Case No.	Citation	Clinical presentation	More than one lesion prior to biopsy	Steroids prior to antiamebic treatment	Antiamebic Treatment (with doses if described)	Increased # lesions after biopsy	Outcome (duration at follow up if given)
		headache, nausea and vomiting			compound Sulfamethoxazole tablets (1.6g, Q6H)		
8	Ono et al. Neuropathology. 2024 (*25*).	76 yo F in Japan with 3 weeks nausea and headache	Yes	Yes, treated as CNS TB initially	Azithromycin, flucytosine, rifampicin, fluconazole	Not clear	Death
9	Atay Ünal et al. Pediatr Infect Dis J. 2024 (*26*)	12 yo M with 1 week of fever, drowsiness, behavioral changes preceded by 1 month of headache, vomiting	No	Yes	Liposomal amphotericin B 5mg/kg<br>Metronidazole 40 mg/kg/day<br>Azithromycin 500 mg IV once daily<br>TMP/SMX 12 mg TMP/kg/day	No	Death
10	Gramp et al. Australas J Dermatol. 2023 (*27*).	66 yo M in Australia with 2 mo of right upper extremity skin lesion followed by fall, seizures, clumsiness and left-sided parasthesia	No	Not described	Albendazole 400 mg BID<br>Fluconazole 200 mg BID<br>Miltefosine 50 mg TID	No	Death
11	Spottiswoode et al. Emerg Infect Dis. 2023 (*28*).	Male in his 50s, presenting with generalized seizure and AMS	No	Yes	Sulfadiazine (1,500 mg PO QID),<br>Fluconazole (1,000 mg [12 mg/kg] PO daily),<br>Flucytosine (3,000 mg [37.5 mg/kg] PO QID),<br>Pentamidine (330 mg [4 mg/kg] IV daily.<br>Azithromycin (500 mg PO daily,<br>Miltefosine (50 mg PO TID),<br>Albendazole (400 mg PO daily)<br><br>Final regimen:<br>Nitroxoline 250mg PO TID, miltefosine, azithromycin, albendazole, fluconazole, and dose-reduced flucytosine	Yes	Survived (15 months)
12	Kim et al. Ann Lab Med. 2022 (*29*).	50 yo M with mild speech disturbance and hemiparesis after focal seizure x3 days	No	Not described	Metronidazole, amphotericin B, sulfadiazine / pyrimethamine	Yes	Death

Case No.	Citation	Clinical presentation	More than one lesion prior to biopsy	Steroids prior to antiamebic treatment	Antiamebic Treatment (with doses if described)	Increased # lesions after biopsy	Outcome (duration at follow up if given)
13	Hirakata et al. BMC Neurol. 2021 (*30*).	60 yo Japanese F with left homonymous hemianopsia	Yes	Yes	Azithrommycin (0.5 g/day), Sulfamethoxazole (1.2 g/day) Trimethoprim (0.24 g/day) Fosfluconazole (0.2 g/day)	Yes	Death
14	Lee et al. Yonsei Med J. 2021 (*31*).	50 yo M with mild speech disturbance and hemiparesis after focal seizure x3 days	No	Yes	"Antiamebiasis medications"	Yes	Death
15	Safavi et al. Am J Trop Med Hyg. 2021 (*32*).	3 yo F from Iran with ataxia, AMS, seizure, left hemiparesis	Yes	Not described	Rifampin, metronidazole, miltefosine	Not clear	Death
16	Suzuki et al. Clin Imaging. 2020 (*33*).	68 yo M with right hemiparesis and seizure	No	Yes	Not described	Yes	Death
17	Piper et al. Int J Infect Dis. 2018 (*34*).	69 yo F with remote breast cancer history with one year of chronic sinus infection leading to saline irrigation with filtered tap water leading to nasal lesions followed by 1 year later left upper extremity focal seizure→ generalized tonic clonic seizure	No	Yes	Miltefosine (50 mg three times daily) Pentamidine (4 mg/kg/day) Sulfadiazine (1.5 g every 6 hours) Flucytosine (37.5 mg/kg every 6 hours) Fluconazole (12 mg/kg/day administered in one dose) Azithromycin (20 mg/kg/day administered in one dose)	Yes	Death
18	Tarai et al. Jpn J Infect Dis. 2018 (*35*).	57 yo M with history of sarcoidosis treated with steroids presented with seizures, AMS	Yes	Yes	Fluconazole 400mg IV daily TMP/SMX 160/800mg BID route not specified Voriconazole 200mg IV BID	Not clear	Death
19	Farnon et al. Clin Infect Dis. 2016 (*36*). CDC. MMWR Morb Mortal Wkly Rep. 2010 (*37*). LaFleur et al. J Radiol Case Rep. 2013 (*38*).	31 yo F with headache, nausea, AMS, seizure with innumerable brain lesions on presentation at post-transplant (renal) day 20.	bilateral cerebral hemispheres, brainstem, cerebellum and right thalamus	Not described	Pentamidine, sulfadiazine, flucytosine, fluconazole, azithromycin, amphotericin, miltefosine	Not clear	Death
20	CDC. MMWR Morb Mortal Wkly Rep. 2010 (*39*).	56 yo M with diabetes, hypertension, liver disease due to hepatocellular carcinoma and fatty liver who presented on PTD (liver) 17 with blurry vision,	Yes	Not described	Sulfadiazine, pyrimethamine, amphotericin	Not clear	Death

Case No.	Citation	Clinical presentation	More than one lesion prior to biopsy	Steroids prior to antiamebic treatment	Antiamebic Treatment (with doses if described)	Increased # lesions after biopsy	Outcome (duration at follow up if given)
		fatigue and ataxia, followed by fever, AMS, fecal incontinence					
21		24 yo M with ESRD due to T1DM and hypertension, kidney pancreas transplant with anti-thymoglobulin treatment who presented on PTD 24 with 5 days of headache, nausea, vomiting, fever	Yes	Not described	Amphotericin, albendazole, fluconazole, azithromycin, and miltefosine	No	Death
22	Greninger et al. Genome Med. 2015 (*40,41*).	15 yo F with insulin dependent diabetes mellitus, celiac disease p/w 7 days right upper extremity weakness, headache, vomiting, ataxia and confusion	Yes	Yes	Azithromycin, sulfadiazine, pentamidine, and flucytosine	Yes	Death
23	Roy et al. Parasitol Res. 2015 (*42*).	11 yo M with 3 weeks of nausea, vomiting, lethargy, clumsiness and right-sided weakness leading to fall; and 4 months of changed handwriting and reading / talking changes and poor performance at school	Yes	Yes	Azithromycin Day(D) 3+58 days 750mg IV daily Fluconazole D3+58 days 400mg IV daily Flucytosine D3+58 days 1000mg to 1425mg pNG q6 hours Pentamidine D3+23 days 160mg IV daily Sulfadiazine 500mg PO / pNG q6 hours D3+58 days Miltefosine 50mg pNG BID D7+26.5 days Amphotericin B10mg/kg/day IV daily D2+3 days Metronidazole D22+39 days 370mg IV q6 hours	Yes	Death
24	Wilson et al. Ann Neurol. 2015 (*43*).	74 yo Chinese F immigrated to US 8 years prior with history of rheumatoid arthritis on hydroxychloroquine who presents with AMS	Yes	Not described	None, broad-spectrum antimicrobial and anti-toxoplasmosis coverage	Yes	Death
25	Tello-Zavala et al. Pediatr Infect Dis J. 2014 (*44*).	4yo M with nausea, vomiting and headache progressing to 2.5 weeks leading to ataxia,	No	Yes	Amphotericin B, dexamethasone	Yes	Death

Case No.	Citation	Clinical presentation	More than one lesion prior to biopsy	Steroids prior to antiamebic treatment	Antiamebic Treatment (with doses if described)	Increased # lesions after biopsy	Outcome (duration at follow up if given)
		abnormal eye movements, dysarthria, with CN palsies, dysmetria and dysdiadochokinesia					
26	Lobo et al. Parasitol Res. 2013 (*45*).	48 yo F from New York with diplopia, slurred speech, headache, cranial nerve palsies	Yes	Yes	No antiamebic therapy; broad-spectrum antimicrobials	Not clear	Death
27	Phillips et al. Clin Neurol Neurosurg. 2013 (*46*).	59yo M with headache, numbness, tingling, dysmetria and dysdiadochokinesia	Yes	Yes	Azithromycin, pentamidine, rifampin, fluconazole and TMP/SMX	No	Death
28	Bando et al. Pathol Int. 2012 (*47*).	68 yo M with progressive right lower extremity hemiparesis x3 months	Yes	Yes	None	Yes	Death
29	Stidd et al. World Neurosurg. 2012 (*48*).	2 yo F with falls for 1.5 weeks, ataxia, irritability and lethargy	Yes	Yes	None	Not clear	Death
30	Yamasaki et al. Neurol Med Chir (Tokyo). 2011 (*49*).	51 yo F with partial seizure progressing to right hemiparesis	No	Yes	No	Yes	Death
31	Ghosh et al. Neurology. 2011 (*50*).	4 yo M with 2 months of headache	No	Not described	Broad-spectrum antimicrobials including amphotericin and fluconazole	Yes	Death
32	Hill et al. Pediatr Neurol. 2011 (*51*).	8 mo old F with lethargy and irritability x4 weeks and then left-sided weakness and left facial palsy	Yes, but atypical infarction lesions	Yes	Anti-TB therapy and anti-fungal therapy including amphotericin B, rifampicin, INH, amikacin and moxifloxacin and IVIG	Not clear	Death
33	Cary et al. Pediatrics. 2010 (*52*).	2 yo M from Kentucky with drowsiness, falls, abnormal eye movements and hyponatremia	Yes	Not described (but treated as CNS TB initially)	Anti-TB therapy prior to *Balamuthia* diagnosis then Pentamidine (4 mg/kg per day) Fluconazole (12 mg/kg per day) Flucytosine (150 mg/kg per day) Sulfadiazine (200 mg/kg per day) Clarithromycin (14 mg/kg per day)	No	Survived (22 months into therapy)
34	Silva et al. Arch Neurol. 2010 (*53*).	47 yo F p/w frontal headache x8d, vomiting x2d followed by	Not clear	Yes	Anti-TB and toxoplasmosis therapy including sulfadiazine and broad-spectrum antimicrobials	Not clear	Death

Case No.	Citation	Clinical presentation	More than one lesion prior to biopsy	Steroids prior to antiamebic treatment	Antiamebic Treatment (with doses if described)	Increased # lesions after biopsy	Outcome (duration at follow up if given)
		nystagmus, ataxia and dysmetria					
35	Kansagra et al. J Neurosurg. 2009 (*54*).	43 yo M with common variable immune deficiency, asplenia with fevers, headache and nausea progressing to vomiting, diplopia, ataxia while on treatment for presumptive fungal meningitis; two months in symptoms progressed to AMS, CNVI palsy, staring spells	Not clear	Not described	Pentamidine, flucytosine, sulfadiazine, nystatin and meropenem	No	Death
36	Perez et al. Ann Diagn Pathol. 2007 (*55*).	40 yo M with alcoholism, hepatitis C, presenting with seizures, headache and myalgias and expressive and receptive aphasia	Yes	Yes	None	Not clear	Death
37	Li et al. Brain Pathol. 2005 (*56*).	6 yo F with gradually worsening headache, stiff neck, nausea and vomiting, and fever x several days	No	Not described	Pentamidine, sulfadiazine, itraconazole and azithromycin	Yes	Death
38	Jung et al. Arch Pathol Lab Med. 2004 (*57*).	72 yo F with focal motor seizure of left face and left hand, transient visual loss and aphasia	Yes	Not described	Pentamidine 300 mg IV daily<br>Sulfadiazine 1500 mg BID<br>Fluconazole 400 mg daily<br>Clarithromycin 500 mg TID	No	Survived (6 mo)
39	Bakardjiev et al. Pediatr Infect Dis J. 2003 (*58*).	2 yo F in California, USA with headache, left abnormal eye movement, papilledema, right hemiparesis	Yes	Yes	TB therapy and broad-spectrum antimicrobials	Not clear	Death
40		7 yo M in California, USA with seizures, ataxia, CNVI palsy	Yes	Yes	Broad-spectrum antimicrobials and antifungals and flucytosine was added with *Balamuthia* diagnosis	Not clear	Death
41		30 mo M in Texas, USA with fever, emesis and ataxia	Yes	Not described	IV pentamidine, metronidazole, fluconazole, amphotericin B and PO itraconazole, flucytosine, azithromycin and sulfadiazine	Not clear	Death

Case No.	Citation	Clinical presentation	More than one lesion prior to biopsy	Steroids prior to antiamebic treatment	Antiamebic Treatment (with doses if described)	Increased # lesions after biopsy	Outcome (duration at follow up if given)
42	Deetz et al. Clin Infect Dis. 2003 (*59*).	64 yo M in California with right hemiparesis and dysarthria	Yes	Yes	TB treatment until biopsy showed ameba then Flucytosine 2g po q6 hours Fluconazole 400mg daily Pentamidine 4 mg/kg/IV q day IV Sulfadiazine 1.5g PO q6 hours Clarithromycin 500mg daily Remained over next 5 years on fluconazole and sulfadiazine	Yes	Survived
43		5 yo F in California with seizures	Yes	Not described	Initially ketoconazole and metronidazole; then Clarithromycin 14 mg/kg/day Flucytosine 110 mg/kg/day Fluconazole 14 mg/kg/day Pentamidine 1mg/kg/day	Not clear	Survived (after 520 days of treatment)
44	Healy JF. AJNR Am J Neuroradiol. 2002 (*60*).	5 yo F presents with GTC seizure	Yes	Not described	"Antiamebic drugs" x16 months	No	Survived as of time of publication
45	Katz et al. Arch Neurol. 2000 (*61*).	52 yo F with idiopathic seizures, chronic neutropenia of unknown cause with progressive lethargy, headaches, and coma	Yes	Yes	Broad-spectrum coverage including amphotericin, fluconazole, TMP/SMX, meropenem, isoniazid, rifampin, ethambutol, pyrazinamide, and metronidazole	No	Death
46	Kodet et al. Pathol Res Pract. 1998 (*62*).	3 yo M with progressive fatigue, seizures and LOC	No	Not described (other than "treated for cerebral edema")	TMP/SMX, amphotericin B, azithromycin, pentamidine	Yes	Death
47	Denney et al. Clin Infect Dis. 1997 (*63*).	32 yo M with nausea, vomiting and lethargy	Yes	Yes	Anti-TB, antistaphylococcal and anti-anaerobic therapy	Not clear	Death
48	Reed et al. Med J Aust. 1997 (*64*).	5 yo M with 18 months of facial skin lesion presented with fever, ataxia and diplopia	Yes	Yes	None	Not clear	Death

Abbreviations used: yo - years old; mo – month; GTC – generalized tonic-clonic; LOC – loss of consciousness; PTD – post transplant day; CN – cranial nerve; TB – tuberculosis; AMS – altered mental status; ESRD – end stage renal disease; T1DM – type 1 diabetes mellitus
TMP-SMX – trimethoprim-sulfamethoxazole; INH – isoniazid; IVIG – intravenous immunoglobulin
PO - per oral; IV – intravenous; IT - intrathecal; pNG – per nasogastric; q – every; d/D – day(s); BID – twice a day; TID – three times a day; QID – four times a day; g – grams; mg – milligrams; kg – kilograms; mg/m2 – milligrams per square meter

References

1. Mei J, Sheng F, Zhang C, Chen X. Imaging monitoring of *Balamuthia* granulomatous amoebic encephalitis. Clin Neurol Neurosurg. 2025;254:108917. PubMed https://doi.org/10.1016/j.clineuro.2025.108917

2. Mei J, Sheng F, Zhang C, Chen X. Corrigendum to "Imaging monitoring of Balamuthia granulomatous amoebic encephalitis". Clin Neurol Neurosurg. 2025;256:109015. PubMed https://doi.org/10.1016/j.clineuro.2025.109015

3. Chen Y, Zhang T, Lou H. Imaging findings of *Balamuthia mandrillaris* amoebic encephalitis in 2 cases with different prognosis and literature review. BMC Neurol. 2025;26:6. PubMed https://doi.org/10.1186/s12883-025-04544-3

4. Zhang J, Jia X, Yang B, Ge Y, Jiang W, Cong Y, et al. Bulk and single-cell transcriptome profiling reveals the dynamic immune response in granulomatous amebic encephalitis caused by *Balamuthia mandrillaris*: a cohort study. Front Immunol. 2025;16:1677014. PubMed https://doi.org/10.3389/fimmu.2025.1677014

5. Teng Z, Liu L, Chen T, Liang J, Yao X, Zhao N, et al. A novel *Balamuthia* lineage causing fatal granulomatous amoebic encephalitis in an immunocompetent infant. Int J Infect Dis. 2025;161:108063. PubMed https://doi.org/10.1016/j.ijid.2025.108063

6. Javed Z, Hussain MM, Ghanchi N, Gilani A, Enam SA. Non-granulomatous meningoencephalitis with *Balamuthia mandrillaris* mimicking a tumor: first confirmed case from Pakistan. Surg Neurol Int. 2024;15:238. PubMed https://doi.org/10.25259/SNI_181_2024

7. Liu J, Zhang W, Wu S, Zeng T, Luo F, Jiang Q, et al. A clinical case report of *Balamuthia* granulomatous amoebic encephalitis in a non-immunocompromised patient and literature review. BMC Infect Dis. 2023;23:245. PubMed https://doi.org/10.1186/s12879-023-08228-6

8. Peng L, Zhou Q, Wu Y, Cao X, Lv Z, Su M, et al. A patient with granulomatous amoebic encephalitis caused by *Balamuthia mandrillaris* survived with two excisions and medication. BMC Infect Dis. 2022;22:54. PubMed https://doi.org/10.1186/s12879-021-07020-8

9. Cuoco JA, Klein BJ, LeBel DP, Faulhaber J, Apfel LS, Witcher MR. Successful treatment of a *Balamuthia mandrillaris* cerebral abscess in a pediatric patient with complete surgical resection and antimicrobial therapy. Pediatr Infect Dis J. 2022;41:e54–7. PubMed https://doi.org/10.1097/INF.0000000000003418

10. Shehab KW, Aboul-Nasr K, Elliott SP. *Balamuthia mandrillaris* granulomatous amebic encephalitis with renal dissemination in a previously healthy child: case report and review of the pediatric literature. J Pediatric Infect Dis Soc. 2018;7:e163–8. PubMed https://doi.org/10.1093/jpids/pix089

11. Lehmer LM, Ulibarri GE, Ragsdale BD, Kunkle J. Cutaneous *Balamuthia mandrillaris* infection as a precursor to *Balamuthia* amoebic encephalitis (BAE) in a healthy 84-year-old Californian. Dermatol Online J. 2017;23:13030/qt8c8720qm. PubMed

12. Kobayashi S, Tsukadaira A, Kobayashi S, Izumiyama S, Yoon HS. Amoebic encephalitis in a farmer. Pathology. 2015;47:720–2. https://doi.org/10.1097/PAT.0000000000000331

13. Krasaelap A, Prechawit S, Chansaenroj J, Punyahotra P, Puthanakit T, Chomtho K, et al. Fatal *Balamuthia* amebic encephalitis in a healthy child: a case report with review of survival cases. Korean J Parasitol. 2013;51:335–41. PubMed https://doi.org/10.3347/kjp.2013.51.3.335

14. Doyle JS, Campbell E, Fuller A, Spelman DW, Cameron R, Malham G, et al. *Balamuthia mandrillaris* brain abscess successfully treated with complete surgical excision and prolonged combination antimicrobial therapy. J Neurosurg. 2011;114:458–62. PubMed https://doi.org/10.3171/2010.10.JNS10677

15. Botterill E, Yip G. A rare survivor of *Balamuthia* granulomatous encephalitis. Clin Neurol Neurosurg. 2011;113(6):499–502. PubMed https://doi.org/10.1016/j.clineuro.2011.01.013

16. Sarica FB, Tufan K, Cekinmez M, Erdoğan B, Altinörs MN. A rare but fatal case of granulomatous amebic encephalitis with brain abscess: the first case reported from Turkey. Turk Neurosurg. 2009;19:256–9. PubMed

17. Tavares M, Correia da Costa JM, Carpenter SS, Santos LA, Afonso C, Aguiar A, et al. Diagnosis of first case of *Balamuthia* amoebic encephalitis in Portugal by immunofluorescence and PCR. J Clin Microbiol. 2006;44:2660–3. PubMed https://doi.org/10.1128/JCM.00479-06

18. Galarza M, Cuccia V, Sosa FP, Monges JA. Pediatric granulomatous cerebral amebiasis: a delayed diagnosis. Pediatr Neurol. 2002;26:153–6. PubMed https://doi.org/10.1016/S0887-8994(01)00360-5

19. Deol I, Robledo L, Meza A, Visvesvara GS, Andrews RJ. Encephalitis due to a free-living amoeba (*Balamuthia mandrillaris*): case report with literature review. Surg Neurol. 2000;53:611–6. PubMed https://doi.org/10.1016/S0090-3019(00)00232-9

20. Degese MF, Prieto MP, Nigro MG, Perazzo J, Pérez Garófalo M, Lemir G, et al. Free-living amoebae infections: case reports identified at a reference parasitology laboratory in Argentina. Acta Parasitol. 2025;70:240. PubMed https://doi.org/10.1007/s11686-025-01198-6

21. Khurana S, Hallur V, Goyal MK, Sehgal R, Radotra BD. Emergence of *Balamuthia mandrillaris* meningoencephalitis in India. Indian J Med Microbiol. 2015;33:298–300. PubMed https://doi.org/10.4103/0255-0857.154887

22. Griesemer DA, Barton LL, Reese CM, Johnson PC, Gabrielsen JA, Talwar D, et al. Amebic meningoencephalitis caused by *Balamuthia mandrillaris*. Pediatr Neurol. 1994;10:249–54. PubMed https://doi.org/10.1016/0887-8994(94)90034-5

23. Edminster SY, Rebbe RW, Khatchadourian C, Hurth KM, Mathew AJ, Huss-Bawab J, et al. The role of plasma metagenomic sequencing in identification of *Balamuthia mandrillaris* encephalitis. Acta Neuropathol Commun. 2025;13:60. PubMed https://doi.org/10.1186/s40478-025-01963-8

24. Liang Y, Wang W. A *Balamuthia* amoebic encephalitis survivor in China, and literature review. Diagn Microbiol Infect Dis. 2025;111:116698. PubMed https://doi.org/10.1016/j.diagmicrobio.2025.116698

25. Ono Y, Higashida K, Yamanouchi K, Nomura S, Hanamatsu Y, Saigo C, et al. *Balamuthia mandrillaris* amoebic encephalitis mimicking tuberculous meningitis. Neuropathology. 2024;44:68–75. PubMed https://doi.org/10.1111/neup.12932

26. Atay Ünal N, Kuzucu P, Bedir Demirdağ T, Aykur M, Günsoy Kiliç Y, Güdeloğlu E, et al. 12-year-old boy with fever, headache and vomiting. Pediatr Infect Dis J. 2024;43:88–90. PubMed https://doi.org/10.1097/INF.0000000000004068

27. Gramp PE, Dooley J, O'Brien B, Jones A, Tan L, Robson J, et al. Fatal granulomatous amebic encephalitis initially presenting with a cutaneous lesion. Australas J Dermatol. 2023;64:e256–61. PubMed https://doi.org/10.1111/ajd.14068

28. Spottiswoode N, Pet D, Kim A, Gruenberg K, Shah M, Ramachandran A, et al. Successful treatment of *Balamuthia mandrillaris* granulomatous amebic encephalitis with nitroxoline. Emerg Infect Dis. 2023;29:197–201. PubMed https://doi.org/10.3201/eid2901.221531

29. Kim JY, Yi MH, Kim M, Yeom JS, Yoo HD, Kim SM, et al. Diagnosis of *Balamuthia mandrillaris* encephalitis by thymine-adenine cloning using universal eukaryotic primers. Ann Lab Med. 2022;42:196–202. PubMed https://doi.org/10.3343/alm.2022.42.2.196

30. Hirakata S, Sakiyama Y, Yoshimura A, Ikeda M, Takahata K, Tashiro Y, et al. The application of shotgun metagenomics to the diagnosis of granulomatous amoebic encephalitis due to *Balamuthia mandrillaris*: a case report. BMC Neurol. 2021;21:392. PubMed https://doi.org/10.1186/s12883-021-02418-y

31. Lee JY, Yu IK, Kim SM, Kim JH, Kim HY. Fulminant disseminating fatal granulomatous amebic encephalitis: the first case report in an immunocompetent patient in South Korea. Yonsei Med J. 2021;62:563–7. PubMed https://doi.org/10.3349/ymj.2021.62.6.563

32. Safavi M, Mehrtash V, Habibi Z, Mohammadpour M, Haghi Ashtiani MT, Sotoudeh Anvari M, et al. Case report: encephalitis caused by *Balamuthia mandrillaris* in a 3-year-old Iranian girl. Am J Trop Med Hyg. 2021;104:1836–40. PubMed https://doi.org/10.4269/ajtmh.20-1257

33. Suzuki T, Okamoto K, Genkai N, Kakita A, Abe H. A homogeneously enhancing mass evolving into multiple hemorrhagic and necrotic lesions in amoebic encephalitis with necrotizing vasculitis. Clin Imaging. 2020;60:48–52. PubMed https://doi.org/10.1016/j.clinimag.2019.10.015

34. Piper KJ, Foster H, Susanto D, Maree CL, Thornton SD, Cobbs CS. Fatal *Balamuthia mandrillaris* brain infection associated with improper nasal lavage. Int J Infect Dis. 2018;77:18–22. PubMed https://doi.org/10.1016/j.ijid.2018.09.013

35. Tarai B, Agarwal P, Krishnamoorthi S, Mewara A, Khurana S. Fatal amoebic meningoencephalitis caused by *Balamuthia mandrillaris* in a sarcoidosis patient. Jpn J Infect Dis. 2018;71:474–6. PubMed https://doi.org/10.7883/yoken.JJID.2018.179

36. Farnon EC, Kokko KE, Budge PJ, Mbaeyi C, Lutterloh EC, Qvarnstrom Y, et al.; Balamuthia Transplant Investigation Teams. Transmission of *Balamuthia mandrillaris* by organ transplantation. Clin Infect Dis. 2016;63:878–88. PubMed https://doi.org/10.1093/cid/ciw422

37. Centers for Disease Control and Prevention (CDC). Notes from the field: transplant-transmitted *Balamuthia mandrillaris*—Arizona, 2010. MMWR Morb Mortal Wkly Rep. 2010;59:1182. PubMed

38. LaFleur M, Joyner D, Schlakman B, Orozco-Castillo L, Khan M. *Balamuthia mandrillaris* meningoencephalitis associated with solid organ transplantation—review of cases. J Radiol Case Rep. 2013;7:9–18. PubMed https://doi.org/10.3941/jrcr.v7i9.1356

39. Centers for Disease Control and Prevention (CDC). *Balamuthia mandrillaris* transmitted through organ transplantation—Mississippi, 2009. MMWR Morb Mortal Wkly Rep. 2010;59:1165–70. PubMed

40. Greninger AL, Messacar K, Dunnebacke T, Naccache SN, Federman S, Bouquet J, et al. Clinical metagenomic identification of *Balamuthia mandrillaris* encephalitis and assembly of the draft genome: the continuing case for reference genome sequencing. Genome Med. 2015;7:113. PubMed https://doi.org/10.1186/s13073-015-0235-2

41. Greninger AL, Messacar K, Dunnebacke T, Naccache SN, Federman S, Bouquet J, et al. Erratum to: Clinical metagenomic identification of *Balamuthia mandrillaris* encephalitis and assembly of the draft genome: the continuing case for reference genome sequencing. Genome Med. 2016;8(1):1. PubMed https://doi.org/10.1186/s13073-015-0257-9

42. Roy SL, Atkins JT, Gennuso R, Kofos D, Sriram RR, Dorlo TP, et al. Assessment of blood-brain barrier penetration of miltefosine used to treat a fatal case of granulomatous amebic encephalitis possibly caused by an unusual *Balamuthia mandrillaris* strain. Parasitol Res. 2015;114:4431–9. PubMed https://doi.org/10.1007/s00436-015-4684-8

43. Wilson MR, Shanbhag NM, Reid MJ, Singhal NS, Gelfand JM, Sample HA, et al. Diagnosing *Balamuthia mandrillaris* encephalitis with metagenomic deep sequencing. Ann Neurol. 2015;78:722–30. PubMed https://doi.org/10.1002/ana.24499

44. Tello-Zavala MC, Bravo-Oro A, Falcón-Escobedo R. Central nervous system infection in an immunocompetent Mexican child. Pediatr Infect Dis J. 2014;33:991, 995–6. PubMed https://doi.org/10.1097/INF.0000000000000379

45. Lobo SA, Patil K, Jain S, Marks S, Visvesvara GS, Tenner M, et al. Diagnostic challenges in *Balamuthia mandrillaris* infections. Parasitol Res. 2013;112:4015–9. PubMed https://doi.org/10.1007/s00436-013-3592-z

46. Phillips BC, Gokden M, Petersen E. Granulomatous encephalitis due to *Balamuthia mandrillaris* is not limited to immune-compromised patients. Clin Neurol Neurosurg. 2013;115:1102–4. PubMed https://doi.org/10.1016/j.clineuro.2012.08.015

47. Bando Y, Takahashi T, Uehara H, Kagegi T, Nagahiro S, Izumi K. Autopsy case of amebic granulomatous meningoencephalitis caused by *Balamuthia mandrillaris* in Japan. Pathol Int. 2012;62:418–23. PubMed https://doi.org/10.1111/j.1440-1827.2012.02816.x

48. Stidd DA, Root B, Weinand ME, Anton R. Granulomatous amoebic encephalitis caused by *Balamuthia mandrillaris* in an immunocompetent girl. World Neurosurg. 2012;78(6):715.e7–12. PubMed https://doi.org/10.1016/j.wneu.2011.10.040

49. Yamasaki K, Sugimoto T, Futami M, Moriyama T, Uehara H, Takeshima H, et al. Granulomatous amoebic encephalitis caused by *Balamuthia mandrillaris*. Neurol Med Chir (Tokyo). 2011;51:667–70. PubMed https://doi.org/10.2176/nmc.51.667

50. Ghosh PS, Ghosh D, Loddenkemper T, Prayson RA, Tekautz T, Sriram CS, et al. Necrotizing granulomatous meningoencephalitis due to *Balamuthia* in an immunocompetent child. Neurology. 2011;77:801–2. PubMed https://doi.org/10.1212/WNL.0b013e31822b0100

51. Hill CP, Damodaran O, Walsh P, Jevon GP, Blyth CC. *Balamuthia* amebic meningoencephalitis and mycotic aneurysms in an infant. Pediatr Neurol. 2011;45:45–8. PubMed https://doi.org/10.1016/j.pediatrneurol.2011.05.003

52. Cary LC, Maul E, Potter C, Wong P, Nelson PT, Given C 2nd, et al. *Balamuthia mandrillaris* meningoencephalitis: survival of a pediatric patient. Pediatrics. 2010 Mar;125(3):e699-703. PubMed https://doi.org/10.1542/peds.2009-1797.

53. Silva RA, Araújo SA, Avellar IF, Pittella JE, Oliveira JT, Christo PP. Granulomatous amoebic meningoencephalitis in an immunocompetent patient. Arch Neurol. 2010;67:1516–20. PubMed https://doi.org/10.1001/archneurol.2010.309

54. Kansagra AP, Menon JP, Yarbrough CK, Urbaniak K, Waters JD, Borys E, et al. *Balamuthia mandrillaris* meningoencephalitis in an immunocompromised patient. Case report. J Neurosurg. 2009;111:301–5. PubMed https://doi.org/10.3171/2008.9.JNS08718

55. Perez MT, Bush LM. Fatal amebic encephalitis caused by *Balamuthia mandrillaris* in an immunocompetent host: a clinicopathological review of pathogenic free-living amebae in human hosts. Ann Diagn Pathol. 2007;11:440–7. PubMed https://doi.org/10.1016/j.anndiagpath.2006.04.003

56. Li Q, Yang XH, Qian J. September 2004: a 6-year-old girl with headache and stiff neck. Brain Pathol. 2005;15:93–5. PubMed https://doi.org/10.1111/j.1750-3639.2005.tb00109.x

57. Jung S, Schelper RL, Visvesvara GS, Chang HT. *Balamuthia mandrillaris* meningoencephalitis in an immunocompetent patient: an unusual clinical course and a favorable outcome. Arch Pathol Lab Med. 2004;128:466–8. PubMed https://doi.org/10.5858/2004-128-466-BMMIAI

58. Bakardjiev A, Azimi PH, Ashouri N, Ascher DP, Janner D, Schuster FL, et al. Amebic encephalitis caused by *Balamuthia mandrillaris*: report of four cases. Pediatr Infect Dis J. 2003;22:447–53. PubMed https://doi.org/10.1097/01.inf.0000066540.18671.f8

59. Deetz TR, Sawyer MH, Billman G, Schuster FL, Visvesvara GS. Successful treatment of *Balamuthia* amoebic encephalitis: presentation of 2 cases. Clin Infect Dis. 2003;37:1304–12. PubMed https://doi.org/10.1086/379020

60. Healy JF. *Balamuthia* amebic encephalitis: radiographic and pathologic findings. AJNR Am J Neuroradiol. 2002;23:486–9. PubMed

61. Katz JD, Ropper AH, Adelman L, Worthington M, Wade P. A case of *Balamuthia mandrillaris* meningoencephalitis. Arch Neurol. 2000;57:1210–2. PubMed https://doi.org/10.1001/archneur.57.8.1210

62. Kodet R, Nohýnková E, Tichý M, Soukup J, Visvesvara GS. Amebic encephalitis caused by *Balamuthia mandrillaris* in a Czech child: description of the first case from Europe. Pathol Res Pract. 1998;194:423–9. PubMed https://doi.org/10.1016/S0344-0338(98)80033-2

63. Denney CF, Iragui VJ, Uber-Zak LD, Karpinski NC, Ziegler EJ, Visvesvara GS, et al. Amebic meningoencephalitis caused by *Balamuthia mandrillaris*: case report and review. Clin Infect Dis. 1997;25:1354–8. PubMed https://doi.org/10.1086/516141

64. Reed RP, Cooke-Yarborough CM, Jaquiery AL, Grimwood K, Kemp AS, Su JC, et al. Fatal granulomatous amoebic encephalitis caused by *Balamuthia mandrillaris*. Med J Aust. 1997;167:82–4. PubMed https://doi.org/10.5694/j.1326-5377.1997.tb138785.x
